# Interpersonal communication competence among nursing students*

**DOI:** 10.1590/1518-8345.3226.3207

**Published:** 2019-10-28

**Authors:** José Luís Guedes dos Santos, Fernanda Hannah da Silva Copelli, Alexandre Pazetto Balsanelli, Caroline Neris Ferreira Sarat, Jouhanna do Carmo Menegaz, Liana Amorim Corrêa Trotte, Marluci Andrade Conceição Stipp, Rafael Marcelo Soder

**Affiliations:** 1 Universidade Federal de Santa Catarina, Departamento de Enfermagem, Florianópolis, SC, Brazil.; 2 Universidade Federal de Santa Catarina, Florianópolis, SC, Brazil.; 3 Scholarship holder at the Conselho Nacional de Pesquisa e Desenvolvimento Científico e Tecnológico (CNPq), Brazil.; 4 Universidade Federal de São Paulo, Departamento de Administração em Serviços de Saúde e Enfermagem, São Paulo, SP, Brazil.; 5 Universidade Federal de Mato Grosso do Sul, Campo Grande, MS, Brazil.; 6 Universidade Federal do Pará, Organização dos Serviços de Saúde, Belém, PA, Brazil.; 7 Universidade Federal do Rio de Janeiro, Escola de Enfermagem Anna Nery, Rio de Janeiro, RJ, Brazil.; 8 Universidade Federal de Santa Maria, Departamento de Ciências da Saúde, Palmeira das Missões, RS, Brazil.

**Keywords:** Professional Competence, Nursing, Education, Communication, Students, Communication Interpersonal, Competência Profissional, Enfermagem, Educação, Comunicação, Estudantes, Comunicação Interpessoal, Competencia Profesional, Enfermería, Educación, Comunicación, Estudiantes, Interpersonal Comunicación

## Abstract

**Objective:**

To identify the level of interpersonal communication competence among nursing students and to correlate its domains with sociodemographic and academic variables.

**Method:**

This is a correlational study, developed through a multicenter research project in six federal universities in Brazil. Data from 1,079 nursing students were collected through a questionnaire with sociodemographic and academic variables and the Interpersonal Communication Competence Scale. Data were analyzed using descriptive and inferential statistics.

**Results:**

The mean of the sum of the Interpersonal Communication Competence Scale was 63.74 (± 7.6). The domains “availability” and “environment control” had, respectively, the highest and lowest averages. There was a statistically significant difference between the sum of the Interpersonal Communication Competence Scale and the variables age, marital status, participation in a research/extension group, and paid professional activity.

**Conclusion:**

This study contributed to identify the level of interpersonal communication competence of nursing students in the Brazilian reality, providing useful information for education in the area.

## Abstract (pt)

**Objetivo:**

identificar o nível de competência de comunicação interpessoal entre estudantes de enfermagem e correlacionar seus domínios com as variáveis sociodemográficas e acadêmicas.

**Método:**

trata-se de um estudo correlacional, desenvolvido por meio de projeto de pesquisa multicêntrico entre seis universidades federais no Brasil. Os dados foram coletados com 1.079 estudantes de enfermagem utilizando um questionário com variáveis sociodemográficas e acadêmicas e da Escala de Competência em Comunicação Interpessoal. Os dados foram analisados por meio de estatística descritiva e inferencial.

**Resultados:**

a média da soma da Escala de Competência em Comunicação Interpessoal foi 63,74 (±7,6). Os domínios “disponibilidade” e “controle do ambiente” apresentaram, respectivamente, as maiores e menores médias. Houve diferença estatística significativa da soma da Escala de Competência em Comunicação Interpessoal com as variáveis idade, estado civil, participação em grupo de pesquisa/extensão e atividade profissional remunerada.

**Conclusão:**

este estudo contribuiu para identificar o nível de competência de comunicação interpessoal de estudantes de enfermagem na realidade brasileira, fornecendo subsídios para a educação na área.

## Abstract (es)

**Objetivo:**

identificar el nivel de competencia comunicativa interpersonal de los estudiantes de enfermería y correlacionar sus dominios con variables sociodemográficas y académicas.

**Método:**

se trata de un estudio correlacional, desarrollado a partir de un proyecto de investigación multicéntrico entre seis universidades federales de Brasil. Se recogieron datos de 1.079 estudiantes de enfermería mediante un cuestionario con variables sociodemográficas y académicas y la Escala de Competencia en Comunicación Interpersonal. Los datos fueron analizados a través de estadísticas descriptivas e inferenciales.

**Resultados:**

el promedio de la suma de la Escala de Competencias en Comunicación Interpersonal fue de 63,74 (±7,6). Los dominios “disponibilidad” y “control del ambiente” presentan, respectivamente, los promedios más altos y los más bajos. Hubo una diferencia estadística significativa en la suma de la Escala de Competencia en Comunicación Interpersonal con las variables edad, estado civil, participación en un grupo de investigación/extensión y actividad profesional remunerada.

**Conclusión:**

este estudio contribuyó en la identificación del nivel de competencia en comunicación interpersonal de los estudiantes de enfermería en la realidad brasileña, proporcionando subsidios para la educación en el área.

## Introduction

Competencies can be defined as behaviors learned during an educational process, which involves the development of knowledge, skills and attitudes for professional practice. Knowledge corresponds to the set of learning and information acquired by individuals, skills are related to the ability to put acquired knowledge into practice, and attitudes are related to the way knowledge is put into practice^(^ 1 ^-^ 2 ^)^.

In Brazil, the five general competencies and skills advocated by the *Diretrizes Curriculares Nacionais* (DCN, National Curriculum Guidelines) for nursing education are decision making, leadership, administration and management, continuing education, and communication^(^ 3 ^)^. Communication stands out among these competences because nurses’ practice is centered on the interpersonal relationship with patients, the nursing team and the multiprofessional team, both to perform care activities and to manage care and health services. In addition, communication permeates and enhances the development and exercise of the other professional nursing skills^(^ 4 ^-^ 5 ^)^. However, many nurses report difficulty in communicating, especially in contexts that constantly require decision making^(^ 6 ^)^.

In this sense, it is important that the development of communication competence permeates the teaching-learning process throughout the nursing course. However, each student experiences this learning differently, considering the knowledge acquired and life-long experiences, which makes it challenging for teachers to create circumstances and teaching strategies to develop communicative skills among nursing students^(^ 7 ^)^.

Given the complexity of developing communication skills, researchers have emphasized the need to use active methodologies and teaching methods that stimulate critical and reflective thinking, based on theoretical and practical integration in nursing^(^ 8 ^-^ 9 ^)^. Despite the importance of competence in interpersonal communication of nursing students, scientific production on this subject is still scarce in Brazil. The available literature mainly includes studies on communication teaching-learning strategies^(^ 8 ^-^ 9 ^)^, which highlights the need for further investigations on this problem^(^ 5 ^)^. Thus, the question is: what is the level of interpersonal communication competence among nursing students? What sociodemographic and academic variables are related to interpersonal communication in nursing students?

This study, developed through a multicenter research, was designed to identify nursing students’ level of interpersonal communication competence and correlate its domains with sociodemographic and academic variables.

## Method

This is a correlational study, developed from a multicenter research project in six federal universities in Brazil: *Universidade Federal de Santa Catarina* (UFSC), *Universidade Federal de Santa Maria* (UFSM), *Universidade Federal do Rio de Janeiro* (UFRJ), *Universidade Federal de São Paulo* (Unifesp), *Universidade Federal do Pará* (UFPA), and *Universidade Federal de Mato Grosso do Sul* (UFMS). So, the research scenarios were the undergraduate nursing courses of the aforementioned universities.

The study population consisted of 1,859 nursing students, from a list obtained from the coordination department of the nursing course of each university. The sampling was of non-probabilistic type, because we expected to apply the instruments for the total population of the study. The inclusion criterion was to be regularly enrolled in the nursing course. Participants who stopped out from college or were on medical leave, or leave of absence of any kind during the data collection phase were excluded. Data were collected in the second term of 2017 and the first of 2018, according to the academic calendar of each institution, during the period of a theoretical class, by prior appointment with the teachers responsible for the subjects. It is emphasized that all classes were surveyed in the same school semester.

The study variables were: dependent (age, sex, marital status, year of graduation, previous degree, technical nursing course, participation in research or extension group, research scholarship, extension scholarship, and paid professional activity ) and independent (competence in interpersonal communication), verified by applying the*Escala de Competência em Comunicação Interpessoal* ^(^ 10 ^)^, which is the validated Brazilian version of the Interpersonal Communication Competence Scale (ICC)^(^ 11 ^)^.

The scale was originally developed in the United States in 1994 to assess an individual’s ability to effectively exchange information with two or more people through verbal and nonverbal communication and language codes. The instrument assesses interpersonal communication as a competence developed from social interactions established by individuals and can be applied to different contexts and situations related to personal life, study and work^(^ 11 ^)^. This is not a specific scale for nursing students, but it has been used with this audience in previous studies with satisfactory results^(^ 12 ^-^ 14 ^)^.

The version validated for use in Brazil in 2014 consists of 17 items, grouped into five domains: environment control, self-disclosure, assertiveness, interaction management, and availability. The domain “environment control” evaluates one’s suitability to an environment to achieve one’s goals. The domain “self-disclosure” represents the ability to demonstrate ideas and thoughts through communication. The domain “assertiveness” assesses firmness and decision in words and attitudes. The domain “interaction management” is related to the handling and interpretation of verbal or nonverbal reactions by the message recipient during the conversation. And the domain “availability” assesses whether the individual is open and available for communication^(^ 10 ^)^.

The ECCI measurement scale consists of a 5-point Likert scale. To get the full score, the items “I have a hard time defending myself” and “It’s hard to find the right words to express myself” are reverse code and need to be recoded. So, for example, score 5 would be marked as 1 in the final score (4 = 2, 3 = 3, 2 = 4, 1 = 5). The total scale score ranges from 17 to 85, and the higher the score, the greater the interpersonal communication ability^(^ 10 ^)^.

Descriptive analyses were performed for all variables. Spearman’s correlation was used to assess the relationship between age and the outcomes assessed. The following tests were used: Student’s t, Anova, Kruskal-Wallis and Mann-Whitney to compare outcome variables between the groups analyzed. For gross analysis, linear regression was used, estimating the gross regression coefficient (β) with its respective 95% confidence intervals (CI).

This study was approved by the Research Ethics Committee of the proposing institution (CAAE: 66306117.9.1001.0121) and also by the other co-participating institutions. The research was developed according to the Resolution of the National Health Council No. 466/2012. All study subjects had their rights secured by signing the Informed Consent Form.

## Results

The study included 1,079 nursing students (58% of the population). The mean age was 22.38 (± 4.7) years. Most were female (86.2%), had no a partner (93.8%), enrolled in the 3rd year of the course (27.1%) and had no previous degree (94.5%).Table 1 presents the complete description of the sample.

**Table 1:** Sociodemographic and academic variables of students, Florianópolis, SC, Brazil, 2017-2018

Sociodemographic and academic variables	Mean	SD*
Age	22.38	4.7
	n	%
Sex (n = 1071)		
Female	923	86.2
Male	148	13.8
Marital Status (n = 1075)		
No partner	1008	93.8
With partner	67	6.2
Year (n = 1077)		
1st year	306	28.4
2nd year	233	21.6
3rd year	292	27.1
4th and 5th years	246	22.8
Previous degree (n = 1079)		
Yes	59	5.5
No	1020	94.5
Nursing technical course (n = 1079)		
Yes	103	9.5
No	976	90.5
Participation in research or extension group (n = 1076)		
Yes	467	43.4
No	609	56.6
Research scholarship (n = 1061)		
Yes	113	10.7
No	891	84.0
Voluntary	57	5.4
Extension scholarship (n = 1061)		
Yes	111	10.5
No	873	82.3
Voluntary	77	7.3
Paid professional activity (n = 1071)		
Yes	121	11.3
No	950	88.7

* Standard deviation

Regarding ECCI, the mean of the sum was 63.74 (± 7.6). The domains “availability” and “environment control” presented, respectively, the highest and lowest means, as shown in Table 2.

**Table 2:** Domains of the Interpersonal Communication Competence Scale. Florianópolis, SC, Brazil, 2017-2018

Domains	Mean	SD*
Environment Control	3.42	0.71
Self-disclosure	3.67	0.72
Assertiveness	3.58	0.66
Interaction management	4.15	0.68
Availability	4.28	0.63

* Standard deviation

In the bivariate analysis, there was a significant difference, regarding the sum, in the mean of the sum in the variables age, marital status, participation in research and extension group, and paid professional activity (Table 3).

**Table 3:** Bivariate analysis between the sum of the Interpersonal Communication Competence Scale and dependent variables. Florianópolis, SC, Brazil, 2017-2018

Dependent variables	ρ*		p-value
Age	0.0805		0.008
	Mean	SD^†^	
Sex			0.278
Female	63.81	7.50	
Male	63.41	8.22	
Marital status			0.003
No partner	63.54	7.67	
With partner	66.80	5.7	
Year			0.425
1st year	63.26	7.94	
2nd year	63.27	7.61	
3rd year	64.57	7.49	
4th and 5th years	63.87	7.19	
Previous degree			0.149
Yes	64.74	8.26	
No	63.69	7.55	
Nursing technical course			0.114
Yes	64.6	7.1	
No	63.65	7.64	
Participation in research or extension group			0.051
Yes	64.54	7.19	
No	63.16	7.83	
Research scholarship			0.071
Yes	65.4	6.55	
No	63.4	7.72	
Voluntary	65.31	7.15	
Extension scholarship			0.932
Yes	64.86	7.67	
No	63.49	7.58	
Voluntary	64.44	7.37	
Paid professional activity			0.044
Yes	64.83	7.12	
No	63.59	7.66	

* Spearman’s correlation coefficient;

† Standard deviation

Regarding the domain “environment control”, a significant difference was observed for age (p-value = 0.025), sex (p-value = 0.007), and marital status (p-value = 0.023). In the domain “self-disclosure”, there was a significant difference only for marital status (p-value = 0.044). Regarding the domain “assertiveness”, there was a significant difference in the variables age (p-value = 0.002) and paid professional activity (p-value = 0.051). In the domain “interaction management”, a significant difference was found in the variables marital status (p-value = 0.054) and research scholarship (p-value = 0.023). Finally, in the domain “availability”, there was a significant difference in the variables sex (p-value = 0.001) and marital status (p-value = 0.003).

In the multiple linear regression analysis, age (β: 0.11; 95% CI: 0.02; 0.21), having a partner (β: 3.26; 95% CI: 1.38; 5.13) and not participating in research and extension group (β: -1.38; 95% CI: -2.29; -0.47) were associated with the ECCI sum (Table 4).

**Table 4:** Multiple linear regression of the variables associated with the sum of the Interpersonal Communication Competence Scale of study participants. Florianópolis, SC, Brazil, 2017-2018

Dependent variables	Gross β* (95% CI)^†^		p-value
Age	0.11(0.02;0.21)		0.021
Sex			0,557
Female	1		
Male	-0.4(-1.72;0.92)		
Marital status			0.001
No partner	1		
With partner	3.26(1.38;5.13)		
Year			0.111
1st year	1		
2nd year	0.01(-1.28;1.30)		
3rd year	1.31(0.09;2.53)		
4th and 5th years	0.62(-0.66;1.89)		
Previous degree			0.298
Yes	1		
No	-1.05(-3.05;0.93)		
Nursing technical course			0.228
Yes	1		
No	-0,95(-2,49;0,59)		
Participation in research or extension group			0.003
Yes	1		
No	-1,38(-2,29;-0,47)		
Research scholarship			0,315
Yes	1		
No	-1.99(-3.48;-0.51)		
Voluntary	-0.08(-2.49;2.33)		
Extension scholarship			0.488
Yes	1		
No	-1.37(-2.86;0.13)		
Voluntary	-0.42(-2.62;1.78)		
Paid professional activity			0.089
Yes	1		
No	-1.25(-2.69;0.19)		

* gross β = gross regression coefficient;

† CI = Confidence interval

In the multiple linear regression analysis of the variables associated with ECCI domains, an association between the “environment control” domain and the mean age variable was identified. There was also an association with males and presence of a partner. The domain “self-disclosure” was associated with non-participation in research and extension group. “Assertiveness” was not associated with any variable. The domain “availability” was less associated with the male sex and more with the presence of a partner.

## Discussion

From a multicenter research in six public higher education institutions, the results of this study present innovative data in the Brazilian context to identify the level of interpersonal communication competence of nursing students. The mean of the sum of ECCI was 63.74 (± 7.6), which can be interpreted as a positive result, since the total ECCI score ranges from 17 to 85, and the higher the score, the greater the ability for interpersonal communication.

Regarding the participant profile shown in Table 1, the sample investigated was composed of young adults, mostly women, single, with no previous college experience, low engagement with research and extension groups, and absence of scholarship. These findings are in line with those found in the literature about the characterization of nursing students^(^ 15 ^)^.

Regarding the sum of the mean of ECCI (63.74 ± 7.6), a similar result was found in undergraduate nursing students from South Korea, where the mean interpersonal communication competence of participants ranged from 52.88 (± 5.02) and 69.94 (± 4.19)^(^ 14 ^)^. This result is positive, since communication, when effective, contributes to nurses’ assertive care^(^ 16 ^-^ 17 ^)^. In addition, communication competence contributes to nurses’ managerial practice, especially for the exercise of leadership^(^ 18 ^)^.

The domains “availability” and “environment control” had, respectively, the highest and lowest means. From these results, it can be inferred that nursing students are accessible and open for communication. This is important because, in the practice of nursing care, there is a need for availability between professional nurse and patient treated^(^ 10 ^)^. In addition, adequate and effective communication contributes to the quality of nursing care, especially in unknown procedures that cause fear and anxiety in patients^(^ 19 ^)^.

In contrast, control over the environment is developed from the professional’s integration in the workspace. In this sense, the lowest mean in this domain represents that it is still necessary for nursing students to express themselves more adequately, aiming at a better adaptation to the environment to improve communication and persuade others around them^(^ 10 ^)^. In addition, this result also reflects the fact that most study participants are first- and second-year students. This stage of education is usually marked by students’ first interactions in care settings of health services, and they are not expected to have control over the professional practice environment.

In the results of the bivariate analysis between the sociodemographic variables of the sample and the result of the sum of ECCI, a significant difference was obtained for age, marital status, participation in research or extension group, and paid professional activity.

The positive association between age and interpersonal communication can be explained by the experiential character related to the learning of communicative competence. Communication is a continuous learning process that develops throughout life. So the accumulated experiences over the years tend to contribute to the development and improvement of communication processes, as well as security in interpersonal relationships^(^ 9 ^)^.

However, it was found that communicative competence does not necessarily increase as the years of undergraduate nursing progress, which makes the relationship between age, communicational competence and year of study paradoxical. It was expected that students in later years would have greater competence than students in early years. Such result may reflect that the skills assessed by the ECCI refer to individual characteristics and not to the training offered specifically in undergraduate nursing courses. However, it also raises reflections on how the interpersonal communication competence is being approached and developed in nursing student education.

In this sense, it is worth highlighting the importance of using teaching strategies that can enhance the development of communication skills in nursing, especially considering that students are entering higher education at increasingly younger ages. Among the active methodologies that can be used, we highlight the potential of clinical simulation to improve nursing students’ communication skills. This strategy enables students to experience situations that require decision making by nurses in health and nursing care settings, whose approach through theoretical classes or traditional teaching methods are not as effective^(^ 20 ^-^ 21 ^)^.

The relationship between a better self-report of interpersonal communication and marital status may be justified by the experience and challenges undergone during a relationship, as already evidenced in a study on nurses’ interpersonal skills in nursing care^(^ 22 ^)^. Couples face important challenges, not only relational problems, but also stress by external factors, and this experience can bring maturation and strengthening of interpersonal communication skills applied to professional practice^(^ 22 ^-^ 23 ^)^.

The influence of participation in a research or extension group in the nursing students’ interpersonal communication competence reinforces the importance of these activities in the formation of critical, reflective and better prepared professionals for the job market. In addition, it is noteworthy that the inclusion of students in research groups meets the curriculum guidelines that advocate the articulation between teaching, research and extension, reflecting benefits to vocational training and science production^(^ 24 ^-^ 25 ^)^.

The relationship between paid professional activity and communication competence can be explained by the skills and attitudes mobilized from inclusion in the labor market. This result is in line with what is expected of nursing graduates, who must have the ability to adapt to the labor market, in view of the competence of communication, in order to meet the DCN demands for the undergraduate nursing course^(^ 26 ^)^.

As a limitation of the study, it can be considered that the variations in size, geographic location and political-pedagogical conception of each institution where the data were collected may have influenced the participants’ responses. In addition, the fact that the proposed instrument is a self-report measure can also be pointed as a study limit, since the participants’ responses are related to their capacity to reflect on their own performance. Further studies should be developed on this subject given the importance of communication skills in the professional practice of nurses.

## Conclusion

The mean of the sum of the Interpersonal Communication Competence Scale in nursing students was 63.74 (± 7.6), which showed a statistically significant difference for the variables age, marital status, participation in research/extension group, and paid professional activity. The important relationship between communication and nurses’ professional practice and the need to develop this competence during undergraduate nursing are emphasized. Communication is a fundamental tool in the teaching and work process of professionals in this area, considering their relationship with the nursing staff, multidisciplinary team, patient and family in health services.
